# Diagnosis of cervical cells based on fractal and Euclidian geometrical measurements: Intrinsic Geometric Cellular Organization

**DOI:** 10.1186/1756-6649-14-2

**Published:** 2014-04-17

**Authors:** Signed E Prieto Bohórquez, Javier O Rodríguez Velásquez, S Catalina Correa Herrera, M Yolanda Soracipa Muñoz

**Affiliations:** 1 Insight Group Researcher. Universidad Militar Nueva Granada, Research Center, Clínica del Country, Cll 45sur 78I 25, Bogotá, Colombia; 2 Insight Group Director. Insight Group. Research Area and Special Internship “Mathematical and Physical Theories Applied to Medicine”, Medicine Faculty, Universidad Militar Nueva Granada, Cr 11 No.101-80, Bogotá, Colombia

**Keywords:** Diagnosis, Fractal, Cervix cancer, Cytology

## Abstract

**Background:**

Fractal geometry has been the basis for the development of a diagnosis of preneoplastic and neoplastic cells that clears up the undetermination of the atypical squamous cells of undetermined significance (ASCUS).

**Methods:**

Pictures of 40 cervix cytology samples diagnosed with conventional parameters were taken. A blind study was developed in which the clinic diagnosis of 10 normal cells, 10 ASCUS, 10 L-SIL and 10 H-SIL was masked. Cellular nucleus and cytoplasm were evaluated in the generalized Box-Counting space, calculating the fractal dimension and number of spaces occupied by the frontier of each object. Further, number of pixels occupied by surface of each object was calculated. Later, the mathematical features of the measures were studied to establish differences or equalities useful for diagnostic application. Finally, the sensibility, specificity, negative likelihood ratio and diagnostic concordance with Kappa coefficient were calculated.

**Results:**

Simultaneous measures of the nuclear surface and the subtraction between the boundaries of cytoplasm and nucleus, lead to differentiate normality, L-SIL and H-SIL. Normality shows values less than or equal to 735 in nucleus surface and values greater or equal to 161 in cytoplasm-nucleus subtraction. L-SIL cells exhibit a nucleus surface with values greater than or equal to 972 and a subtraction between nucleus-cytoplasm higher to 130. L-SIL cells show cytoplasm-nucleus values less than 120. The rank between 120–130 in cytoplasm-nucleus subtraction corresponds to evolution between L-SIL and H-SIL. Sensibility and specificity values were 100%, the negative likelihood ratio was zero and Kappa coefficient was equal to 1.

**Conclusions:**

A new diagnostic methodology of clinic applicability was developed based on fractal and euclidean geometry, which is useful for evaluation of cervix cytology.

## Background

Uterine cervical cancer is the second most common cancer in women worldwide and accounts for about 20% of all gynecological cancers [1]. It has a mortality comparable to breast cancer, with 250,000 deaths annually and about 500,000 new cases reported each year [2]. This disease can be prevented through the cervico-vaginal cytology (CCV), which can detect abnormal cervical tissue before it progresses to invasive cancer [3]. Its diagnosis in the early stages is very important [4,5] as it can be curable [6-8] and the prognosis of patients have a survival rate at 5 years above 90%, which makes the CCV an essential tool for reducing mortality [9].

CCV usually presents high specificity ranging from 80% to 98%, but it has sensitivity highly variable with rates between 45% and 85% [10]. A systematic review by Nanda and colleagues to assess the diagnostic accuracy of the CCV, found an average sensitivity of 51% and a specificity of 98% [11]. Also, false negatives are presented with a prevalence between 20% and 40% [12,13], with an average of 35.5% [14]. The CCV has also demonstrated a lower specificity for high grade intraepithelial lesions than for low-grade lesions [15].

The difficulties in achieving higher sensitivity and specificity values are largely associated to the fact that assessments are based on qualitative parameters, which involves intra and inter-observer discrepancies. Also there are difficulties to diagnose cells with characteristics close to the limits from one to another state. Even with the adequate samples and expert pathologists, the interobserver variability still reduces cytological diagnosis accuracy [16,17].

As a result of these problems, it has not been established a unified global assessment. Currently the most widely used system for reporting CCV is the Bethesda System [18], which presents a narrative report that includes all cytology aspects (hormonal, morphological and microbiological). In this system the cells are classified as normal, Atypical Squamous Cells of Undetermined Significance (ASCUS), low-grade squamous intraepithelial lesion (L-SIL) or high grade squamous intraepithelial lesion (H-SIL). The H-SIL state includes the states before known as moderate dysplasia, severe dysplasia and carcinoma in situ, or CIN 2 and CIN 3 [19].

ASCUS category was introduced to define more clearly the “gray zone” between benign and malignant lesions, being the category with the lowest inter-observer reproducibility and the greater diagnostic challenge [20-22].

The problems in the objective measurement of the cellular structure are associated with failure to have methodologies based on objective and reproducible measures of its irregularity, in the clinical practice. Usually, when steps are performed to objectify medical observations, Euclidean geometry is used. However, it was developed for the measurement of regular figures such as lines, areas or volumes. It has been shown that the use of regular measurements in irregular objects may lead to paradoxical results [23].

Fractal geometry was developed to measure the irregularity of the objects, with a dimensionless numerical measure called fractal dimension [23,24]. Methodologies for the determination of the fractal dimension depend on the type of object being measured. In the case of mathematical fractals such as the Sierpinski triangle, the dimension of Haussdorff is used. The statistical fractals are characterized by hyperbolic distributions of the variables, and are measured by the Zipf/Mandelbrot fractal dimension. Structures that have overlapping parts, such as the anatomical structures of the human body and chaotic attractors, are evaluated with Box Counting dimension [24], which relates to the spatial occupation of an object at different scales.

In order to develop objective and reproducible diagnostic measures in cervical cells, Rodriguez et al. have used fractal geometry, differentiating normality and L-SIL by means of the concepts of Intrinsic mathematical Harmony (IMH) and variability of the fractal dimension [25,26]. These concepts allow to evaluate objectively and quantitatively the cells classified as ASCUS, establishing if they have values close to normality or L-SIL, overcoming the difficulties caused by the use of qualitative parameters, such as the Bethesda system.

Recently it has been developed methodologies to characterize different fractal structures from fractal and Euclidean simultaneous measures, allowing the differentiation between normality and disease with clinical application. Such is the case of a method that calculates all possible coronary arteries in the process of restenosis, from normality to total occlusion of the lumen [27]. This method allows to quantify the degree of development of restenosis and thus improves measurements made only with fractal geometry [28]. Similarly, a fractal-euclidean methodology allows to distinguish normal from abnormal erythrocytes, being useful for determining the viability of bags for transfusion [29].

Following this new research perspective, useful in medical practice, the objective of this work is to apply euclidean and fractal geometries to develop a diagnostic method of clinical application for preneoplastic and neoplastic lesions of the cervix.

## Methods

### Definitions

#### Fractal

Term derived from the Latin fractus (broken). It was proposed by Benoit Mandelbrot to refer to a fragmented or irregular object which structure is repeated at different scales [24]. More accurately, a fractal is defined as a set for which the Hausdorff/Besicovitch dimension strictly exceeds the topological dimension [30].

#### Fractal dimension

Numerical measure that evaluates the irregularity of an object. The essential mathematical property of a fractal object is that its fractal dimension is a non-integer. There are different methods to calculate fractal dimensions, depending on the characteristics of the object. In the case of wild fractals, such as cervical cells, the definition of Box-Counting dimension is used [24].

#### Box-counting dimension

The Box-Counting dimension allows to quantify the changes in the irregularity of an object at different scales. For this purpose are used grids of different sizes which are overlapped to the object, in order to count the spaces occupied by the object in the different grids [24]. The values obtained are used in the next equation, to obtain the fractal dimension:

(1) $$\begin{array}{ll} D & =\frac{\mathrm{LogN}\left(2^{-\left(K+1\right)}\right)-\mathrm{LogN}\left(2^{-K}\right)}{\mathrm{Log}2^{K+1}-\mathrm{Log}2^{K}}\\ & =\mathrm{Lo}g_{2}\frac{N\left(2^{-\left(K+1\right)}\right)}{N\left(2^{-K}\right)} \end{array}$$

Where:

N: Number of squares containing the outline of the object.

K: Level of partition in the grid.

D: fractal dimension.

### Object surface

**Figure 1:**
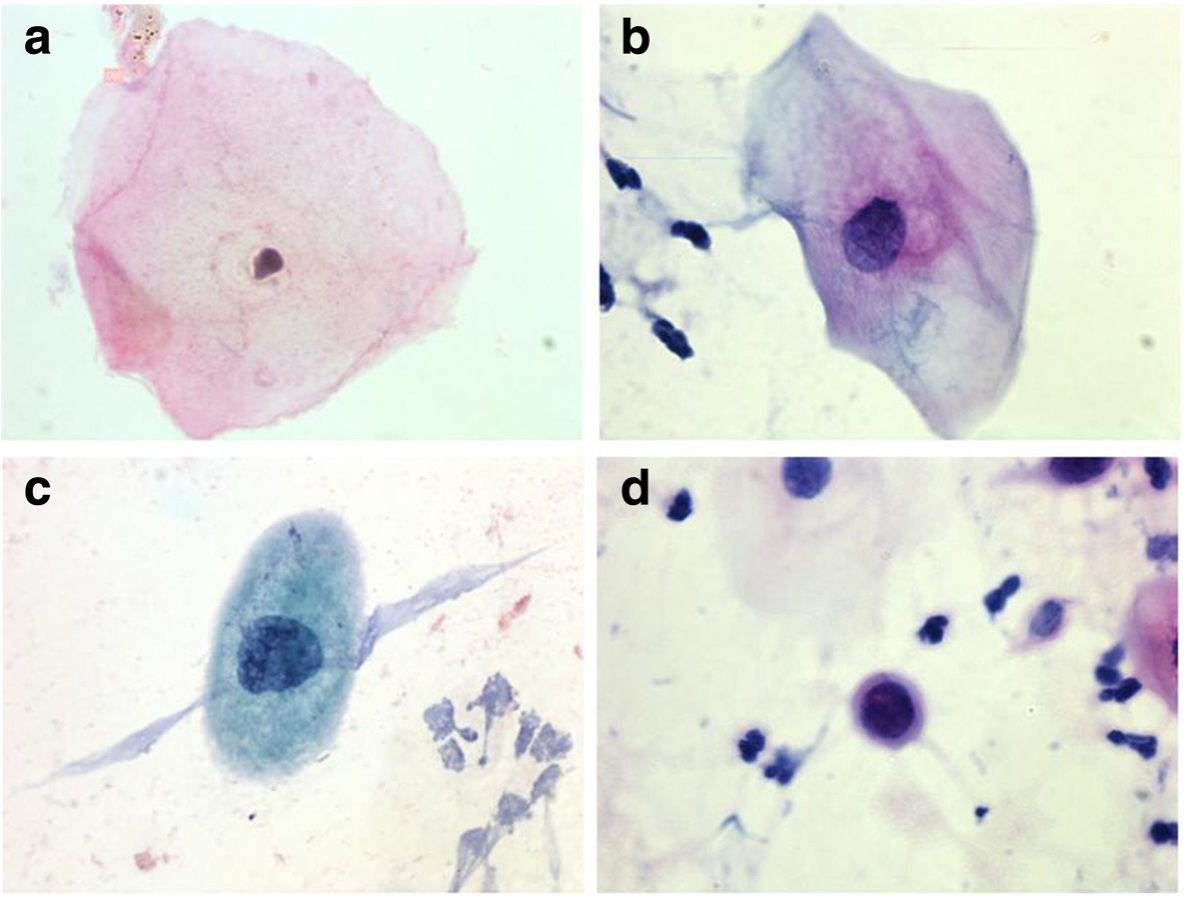
**Examples of the measured cells. a**. Normal cell. **b**. ASCUS cell. **c**. L-SIL cell. **d**. H-SIL cell.

**Figure 2:**
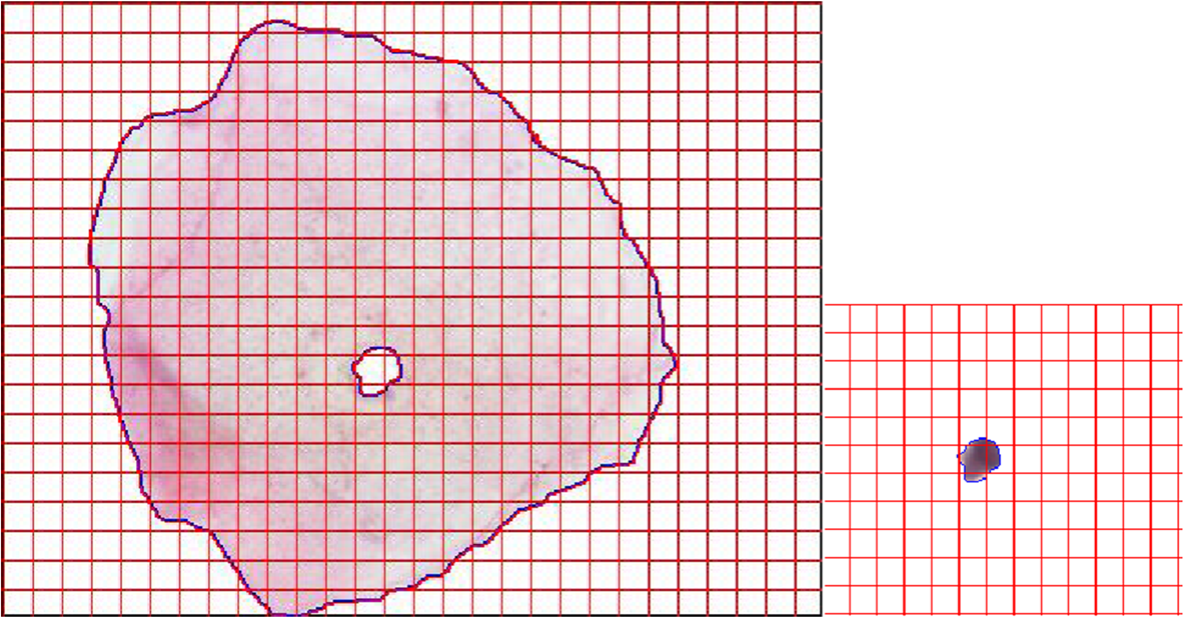
**Cytoplasm (left) and nucleus (right) surfaces of a normal cell, shown as an example of the objects surfaces measured.** For each object, the software applied counts the number of pixels occupied inside the blue line, including the pixels of the border. In the case of the cytoplasm, it counts the number of pixels included between the two blue lines: the first one corresponding to the cytoplasm border, and the other one, corresponding to the nucleus border.

Number of pixels occupied by each one of the objects defined in each cell (nucleus and cytoplasm). See Figures 1 and 2.

### Object frontier

**Figure 3:**
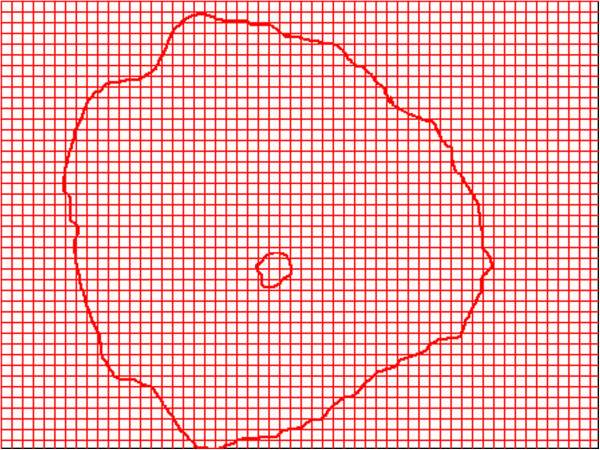
**Example of the frontiers of the same normal cell shown in Figure **2**.** The software counts the number of squares touched by the red lines, with the grid of 2 pixel’s side, the big one corresponding to the cytoplasm border, and the little one, corresponding to the nucleus border.

Number of squares occupied by each one of the contours of the objects defined in each cell (nucleus and cytoplasm), with the grid of 2 pixel’s side. See Figure 3.

### Substraction of Cytoplasm-Nucleus frontiers

Subtracting the values of the frontier of the cytoplasm and the nucleus. For example, if it was obtained a value of 420 in the cytoplasm frontier, and 35 in the nucleus frontier, these two values are subtracted, so the substraction of Cytoplasm-Nucleus frontiers will be 385. See Tables 1, 2, 3 and 4.

**Table 1:** Measurements of the normal cells

**Cell**	**Nucleus**		**Citoplasm Frontier**	**Subtraction of Cytoplasm-Nucleus frontiers**
	**Frontier**	**Surface**		
**1**	35	299	420	385
**2**	39	325	400	361
**3**	41	355	487	446
**4**	41	340	389	348
**5**	42	336	502	460
**6**	42	349	485	443
**7**	42	367	548	506
**8**	43	352	362	319
**9**	45	413	523	478
**10**	55	735	216	161

The frontier is expressed in number of squares in the grill of 2 pixels, and the surface in pixels.

**Table 2:** Measures of the ASCUS cells

**Cell**	**Nucleus**		**Citoplasm Frontier**	**Subtraction of Cytoplasm-Nucleus frontiers**
	**Frontier**	**Surface**		
**1**	72	1283	205	133
**2**	79	1530	476	397
**3**	86	1773	485	399
**4**	87	1887	355	268
**5**	95	2318	545	450
**6**	99	2151	482	383
**7**	104	2449	430	326
**8**	107	2776	322	215
**9**	129	4023	462	333
**10**	139	4765	479	340

The frontier is expressed in number of squares in the grill of 2 pixels, and the surface in pixels.

**Table 3:** Measures of the L-SIL cells

**Cell**	**Nucleus**		**Citoplasm Frontier**	**Subtraction of Cytoplasm-Nucleus frontiers**
	**Frontier**	**Surface**		
**1**	69	972	301	232
**2**	100	2406	413	313
**3**	104	2306	526	422
**4**	107	2311	418	311
**5**	109	2833	229	120
**6**	114	2883	235	121
**7**	124	3385	330	206
**8**	141	4057	334	193
**9**	159	5564	439	280
**10**	127	2936	290	163

The frontier is expressed in number of squares in the grill of 2 pixels, and the surface in pixels.

**Table 4:** Measures of the H-SIL cells

**Cell**	**Nucleus**		**Citoplasm Frontier**	**Subtraction of Cytoplasm-Nucleus frontiers**
	**Frontier**	**Surface**		
**1**	48	686	160	112
**2**	51	666	160	109
**3**	53	520	93	40
**4**	68	952	142	74
**5**	77	1194	129	52
**6**	78	1356	113	35
**7**	82	1458	212	130
**8**	97	2231	169	72
**9**	119	2706	174	55
**10**	63	870	120	57

The frontier is expressed in number of squares in the grill of 2 pixels, and the surface in pixels.

### Procedure

Cervix cytology samples of 40 women between 20-55 years were selected from the Insight Group data base. These samples show reports of normal cytology and different grades of lesion including carcinoma, issued by an expert pathologist according to conventional parameters, where carcinoma cells are included in H-SIL classification [19]. Digital pictures of 10 normal cells, 10 ASCUS, 10 L-SIL and 10 H-SIL were taken from cervical smear on the glass slides which were observed via Leika DM-2500 optical microscope with a 100X zoom. The pictures were carried to a computer interface, in order to be analyzed through an image editor, masking the diagnostic conclusion of cytologies.A physical–mathematical induction was developed, starting from the evaluation of two defined mathematical objects, which are the nucleus and the cytoplasm without nucleus of each cell (which correspond to the nucleus and the cytoplasm traditionally observed, but evaluated in the Box Counting space). Next the fractal dimension of each defined object is calculated. For this purpose it was used a previously developed software where each image is superimposed with two grids of 2 and 4 pixels. Then the number of squares containing the contours of each object is counted in each grid, obtaining different values for the nucleus and the cytoplasm (see Figure 3). Then, a plot of the logarithm of the number of squares containing the outline of the object versus the logarithm of the level of partition in the grid is made. The slope of the line relating these two variables (derivative), with the inverted signal, is the dimension of box counting that can be defined through the following expression: Equation 1.Later, the number of squares occupied by the border of each object with grill of 2 pixels (see Figure 3) and the number of pixels occupying the surface of the defined objects (see Figure 2) were calculated. Finally, mathematical equalities and differences were searched, looking for characteristics of normality and disease as well as the evolution states between normality and disease, developing the geometric diagnosis without knowledge of conventional diagnosis.

### Ethics statement

Present research was undertaken following the provisions of Declaration of Helsinki in 1995, due to this methodology have a theoretical character based on non-invasive test previously prescribed. The patient’s privacy, integrity and anonymity were protected. For those reasons, the local ethics committee was consulted and deemed the work exempt from needing full ethical approval.

### Consent statement

Because the present research have a theoretical character and is based on non-invasive test previously prescribed, informed consent was not necessary.

### Statistical analysis

After developing the physical and mathematical diagnostic parameters, the diagnostic of each cell was established. Then, the clinical diagnostics of cytologies, evaluated with Bethesda System by the expert, were unmasked, and were taken as the Gold-Standard. Since the physical–mathematical diagnosis differentiate between normality and disease, for comparison with the Gold Standard, cells conventionally classified as L-SIL or H-SIL were listed within a single classification as sick, allowing to establish a contingency Table 2 * 2 to compare the number of normal and pathological cases concordant and non-concordant. Then sensibility, specificity and negative likelihood ratio were calculated. The level of concordance between Gold-Standard and physical–mathematical diagnosis was evaluated through Kappa coefficient.

The cells classified as ASCUS were excluded of the statistical analysis because they don’t have a specific diagnosis of normality or disease from Gold-standard. However, starting from the physical–mathematical evaluations, diagnostic relations of these cells were sought respect to the normality and disease states in order to specify quantitative differentiations.

## Results

The nucleus surface in pixels showed values between: 299 and 735 for normality; 1283 and 4765 for ASCUS; 972 and 5564 for L-SIL and 520 and 2706 for H-SIL. These values can differentiate normality, ASCUS and L-SIL. However they do not differentiate normality and H-SIL (Tables 1, 2, 3 and 4).

Measurements of the cytoplasm frontier in grid of 2 pixel’s side ranged between: 216–548 for normal cells; 205–545 for ASCUS cells; 229–526 for L-SIL; 93–212 for H-SIL cells.

Measures of the nucleus frontier in grid of 2 pixel’s side presented values between: 35 and 55 for normal cells; 72 and 139 for ASCUS; between 69 and 159 for L-SIL and 48 and 119 for H-SIL.

Subtracting the measured values of the nucleus and cytoplasm frontiers showed values between 161 and 506 for normality, between 133 and 450 for ASCUS, 120 to 422 for L-SIL and between 35 and 130 for H-SIL.

These measures of Substraction of Cytoplasm-Nucleus frontiers mathematically differentiate normal cells from the H-SIL. Additionally there is an overlap in the values of L-SIL and H-SIL frontiers in grid of 2 pixel’s side, however when analyzing the results, it is found that only the No. 7 H-SIL cell has values within the range of L-SIL, showing the value of 130 while the remaining H-SIL cells feature values less than or equal to 112. Similarly, it is noted that only two L-SIL cells show values within the range of H-SIL, corresponding to 120 and 121 respectively (Tables 3 and 4). In the conventional diagnostic classifications it is found a diffuse space or “gray area” between L-SIL and H-SIL classifications; with this methodology is mathematically possible to quantify all the evolution between normality and disease. All the ASCUS cells in this study behaved mathematically as L-SIL cells.

The fractal dimensions of the objects were calculated and the results are shown in Table 5. Statistical analysis resulted in sensitivity and specificity of 100%, a likelihood ratio of negative zero, and a Kappa coefficient of 1.

**Table 5:** Minimum (MIN) and maximum (MAX) fractal dimensions found in each group for the measured objects

	**Fractal dimension**			
**Dx.**		**N**	**C**	**T**
**NORMAL**	**MIN**	0,8590	0,9547	0,9607
	**MAX**	1,0984	0,9943	1,0085
**ASCUS**	**MIN**	0,9316	0,9342	0,9591
	**MAX**	0,9717	1,0249	1,0203
**L-SIL**	**MIN**	0,9235	0,9631	0,9603
	**MAX**	1,0305	1,0155	1,0659
**H-SIL**	**MIN**	0,9229	0,9123	1,0438
	**MAX**	1,1460	1,0570	1,2674

N: Nucleus. C: Citoplasm. T. Total object.

### Mathematical–physical diagnosis

Normal cells are characterized by nuclear surfaces less than or equal to 735 pixels and values greater than or equal to 161 squares in the grid of 2 pixel’s side in the rest of the frontiers Cytoplasm-Nucleus.

L-SIL cells have values greater than or equal to 972 pixels on the nucleus surface and a value greater than 130 squares in the grid of 2 pixel’s side in the rest of Cytoplasm-Nucleus frontiers.

H-SIL cells are characterized by values less than 120 in the rest of Cytoplasm-Nucleus frontiers.

The differentiation between L-SIL and H-SIL cells is done only with the subtraction of Cytoplasm-Nucleus frontiers. The range 120–130 squares in the grid of 2 pixel’s side in the subtraction of Cytoplasm-Nucleus frontiers corresponds to the evolution between L-SIL and H-SIL cells.

## Discussion

This is the first work in which a fractal and euclidean diagnosis of cervical cells, observed in cytologies ranging from normality to H-SIL, is done. This is a diagnostic tool with clinic applicability, that determine the lesion grade of cells in an objective and reproducible way. Quantitative differences between normality, L-SIL and H-SIL were established, quantifying the increase in lesion severity from measures of cellular occupation in generalized Box-Counting space. A mathematical order underlying to cellular structure in preneoplastic and neoplastic development was evidenced, allowing overcoming reproducibility difficulties of the current classification systems, such as Bethesda System. In order to determine the cellular state, the conventional classification methodologies use the observation of the nucleus and the cytoplasm [13,16], as well as the simultaneous observation of qualitative parameters; however, these show reproducibility problems [11,16,17,19]. This methodology allows quantifying the increase of the nuclear frontier and surface in an objective way, taking in account its irregular character with fractal methods.

The measure of the difference between nucleus-cytoplasm frontiers shows the stage in which the cell is, and besides quantifies how close it is to a higher severity stage. This is useful in the diagnostic discrimination of ASCUS cells because it can clarify how close they are to normality or disease, as well as their possible evolution. So, the diagnostic problems of this qualitative classification [19-22] have been resolved by means of the underlying harmony to cellular structure, which was shown in the established mathematical measures. So, it was possible to quantify the differences and similarities of the cells even when the clinic diagnostics were masked. The confidence in a mathematical and geometric order gave birth to this reproducible and objective result, thus giving a solution useful to unify the qualitative systems of cytology classification.

The diagnostic capability of the developed method was possible thanks to the simplicity of the mathematical language, because it showed that it was a simple phenomenon in so far the problem was observed from a physical and mathematical perspective. Also it showed that the problem evaluation had been complicated because of the type of observation and the qualitative language conventionally used, which is based on classifications from traditional medicine. For this reason, although there was evidence of the association between normality and disease to different relations among nucleus and cytoplasm dimensions, it was not possible to establish such differences due to descriptive and qualitative language of conventional medicine. From the mathematical language the phenomenon is observed as a totality and all the possibilities in the evolution process can be obtained without the use of qualitative classifications which lose the phenomenon generality. So, normality and disease are particular geometric states within the whole phenomenon, in the same way as theoretical physics, where a single mathematical expression includes a whole phenomenon [31,32]. For this reason, this kind of perspective of research is independent of epidemiological and statistical considerations, focusing on universal proportions to account for the whole phenomenon, being applicable to each particular case, and not just a majority population. This type of physical–mathematical approach makes unnecessary to start from the study of many cases to achieve diagnostic conclusions applicable to clinic. The statistical analysis of this study to evaluate the diagnostic capability of method was performed in order to meet the current requirements of medical research, showing the best Concordance with the Gold Standard, with a sensitivity and specificity of 100%, a negative likelihood ratio of zero, and a Kappa coefficient of 1.

ASCUS cells evaluated in this study didn’t show quantitative features of normality; however there is evidence [25,26] that is not always so. Is very important to develop applications of this method to a greater number of cells in order to confirm the obtained limits and thereby refine its clinic applicability. Nevertheless, because of the mathematical and theoretical character of this method, these limits can vary without affecting the general diagnosis.

The value of fractal dimension, observed in isolation, is not sufficient to establish diagnostic differences [33]. This makes it necessary to include a new measure, in this case from Euclidean geometry, but in the context of Box Counting space, thus respecting the irregularity of the object. For this, the values of the nucleus and cytoplasm borders and the surface were observed, which are a quantification of its length, constituting a Euclidean magnitude in a fractal context.

Thus, unlike other studies in medicine in which Euclidean geometry applies regardless of the irregularity of the object, in this work, simultaneous Euclidean and fractal measures are achieved. Such simultaneous measurement had already been applied in the analysis of coronary restenosis process [27] and erythrocyte morphophysiology [29], differentiating normal and abnormal states.

The developed method is more practical than the conventional ones because it is applicable to each particular case without depending on neither population analysis nor risk factors. Moreover, it is a quantitative method that makes itself more objective and reproducible compared with the current qualitative classifications. With this methodology it is possible to facilitate the creation and evaluation of more effective and economical public health policies [10,34,35], for a more detailed monitoring over time of patients with cytologies that present some kind of squamous epithelial cell abnormality.

According to the hypothesis of genetic cancer etiology, the tumors appear as a consequence of clonal expansion of a single cell with a genetic alteration, therefore implying a disruption of the nucleus for the tumoral development [36]. In this sense it might be thought that the obtained result supports this hypothesis, as it is based in a morphometric measurement of the nucleus alteration. However, regardless of the veracity or falsity of this statement, this methodology is based on a non-causal physical and mathematical thought, because establishes diagnostic differences independently of any consideration regards to the origin of cancer development. Thus, this type of measure could be useful for objective and quantitative evaluation of preneoplastic and neoplastic cells from other tissues.

Following the non-causal perspective of modern physics [37-39], the present methodology was developed from a point of view where cause-effect relations were not considered; this is why it is independent of external factors such as age, risk factors and any population analysis. Here, only temporal windows [40] of the cells are observed, revealing a harmonic order underlying to cell structure, thus establishing mathematical characteristics to differentiate among each one of the grades of intraepithelial lesions. From this non-causal perspective, also there have been developed predictions and methodologies of diagnostic help in other areas of medicine, such as cardiology [31,32,41,42], immunology [43], molecular biology [44], epidemics prediction, [45] and infectology [46,47], among others.

## Conclusions

Starting from fractal and euclidean measures, it was developed a new diagnostic method for cervical cells observables in cervix cytology. The method is useful for differentiate in an objective and reproducible way, normal, L-SIL and H-SIL cells at clinical level. It was based on a mathematical order subjacent to cellular structure, where the increase of the nuclear frontier and surface evaluated in the generalized Box-Counting space, allows to quantify the level of progress of the lesion. It constitutes a solution to the problems of reproducibility associated to the current classification systems, such as the Bethesda.

## Competing interests

The authors declare that they have no competing interests.

## Authors’ contributions

SEP conceived the study, and participated in its design and coordination, JOR conceived the study, and participated in its design and coordination, SCC developed software and mathematical - fractal calculations and drafted the document, MYS participated in data recollection and its systematization.

## Pre-publication history

The pre-publication history for this paper can be accessed here:

http://www.biomedcentral.com/1756-6649/14/2/prepub
